# The Maternal and Infant Environmental Health Riskscape study of perinatal disparities in greater Houston: rationale, study design and participant profiles

**DOI:** 10.3389/frph.2024.1304717

**Published:** 2024-04-22

**Authors:** Elaine Symanski, Kristina W. Whitworth, Hector Mendez-Figueroa, Kjersti M. Aagaard, Iman Moussa, Juan Alvarez, Adrien Chardon Fabian, Kurunthachalam Kannan, Cheryl L. Walker, Cristian Coarfa, Melissa A. Suter, Hamisu M. Salihu

**Affiliations:** ^1^ Center for Precision Environmental Health, Baylor College of Medicine, Houston, TX, United States; ^2^ Section of Epidemiology and Population Sciences, Department of Medicine, Baylor College of Medicine, Houston, TX, United States; ^3^ Division of Maternal-Fetal Medicine, Department of Obstetrics, Gynecology and Reproductive Sciences, McGovern Medical School at UTHealth, Houston, TX, United States; ^4^ Division of Maternal-Fetal Medicine, Department of Obstetrics & Gynecology, Baylor College of Medicine & Texas Children’s Hospital, Houston, TX, United States; ^5^ New York State Department of Health, Wadsworth Center, Albany, NY, United States; ^6^ Department of Molecular and Cell Biology, Baylor College of Medicine, Houston, TX, United States; ^7^ Department of Family and Community Medicine, Baylor College of Medicine, Houston, TX, United States

**Keywords:** MIEHR, environment, health disparities, maternal health, preterm birth, neighborhood, stress

## Abstract

**Introduction:**

The Maternal and Infant Environmental Health Riskscape (MIEHR) Center was established to address the interplay among chemical and non-chemical stressors in the biological, physical, social, and built environments that disproportionately impact perinatal health among Black pregnant people in a large and diverse urban area with documented disparities in the U.S.

**Methods:**

The MIEHR cohort is recruiting non-Hispanic Black and non-Hispanic white pregnant people who deliver their infants at major obstetric hospitals in Houston, Texas. At enrollment, all participants are asked to provide urine samples for chemical [metals, cotinine, and polycyclic aromatic hydrocarbons (PAHs)] analyses and blood samples. A subset of the cohort is asked to provide oral and vaginal swabs, and fecal samples. Questionnaire and electronic health record data gather information about residential address history during pregnancy, pregnancy history and prenatal care, sociodemographic and lifestyle factors, experiences of discrimination and stress, and sources of social support. Using information on where a participant lived during their pregnancy, features of their neighborhood environment are characterized. We provide summaries of key individual- and neighborhood-level features of the entire cohort, as well as for Black and white participants separately.

**Results:**

Between April 2021 and February 2023, 1,244 pregnant people were recruited. Nearly all participants provided urine samples and slightly less than half provided blood samples. PAH exposure patterns as assessed on 47% of participants thus far showed varying levels depending on metabolite as compared to previous studies. Additionally, analyses suggest differences between Black and white pregnant people in experiences of discrimination, stress, and levels of social support, as well as in neighborhood characteristics.

**Discussion:**

Our findings to date highlight racial differences in experiences of discrimination, stress, and levels of support, as well as neighborhood characteristics. Recruitment of the cohort is ongoing and additional neighborhood metrics are being constructed. Biospecimens will be analyzed for metals and PAH metabolites (urine samples), miRNAs (plasma samples) and the microbiome (oral swabs). Once enrollment ends, formal assessments are planned to elucidate individual- and neighborhood-level features in the environmental riskscape that contribute to Black-White disparities in perinatal health.

## Introduction

Despite major breakthroughs in medical care, health inequities persist among U.S. populations and are especially consequential for pregnant people and their children. As compared with other racial and ethnic groups, Black pregnant people suffer the highest risks of poor pregnancy outcomes in the nation. Essentially unchanged from the period 2007 to 2016 (1), pregnancy-related mortality in 2020 was almost 3 times higher among Black as compared to white pregnant people (2). There are also disparities in the prevalence of preterm birth, which is a primary cause of perinatal death and a risk factor for adverse health outcomes for an infant throughout the life course (3), with a prevalence of 14.4% and 9.1% among Black and non-Hispanic white populations, respectively (4). Similar to national trends, racial inequities in health outcomes are strikingly evident in Harris County, Texas (5), the third most populous county in the nation and home to Houston, a city with an immensely diverse population and more families living below the poverty line than the rest of Texas or the nation (6). Pointedly, Houston and Harris County both earned an “F” in the March of Dimes 2022 Report Card for preterm birth (7).

Though not well-understood, racial disparities in perinatal health are likely related to factors other than genetics, behavior, access to health care or individual-level socioeconomic status (8–12). Indeed, the American College of Obstetricians and Gynecologists (ACOG) recognizes the importance of structural racism (i.e., macro-level conditions that limit opportunities, resources, and well-being of less privileged groups) on influencing maternal and infant health outcomes (13). Because of redlining and other exclusionary practices of financial lenders, Black communities have been historically burdened by housing discrimination and neighborhood segregation (14), leading to limited investments in communities of color including grocery stores, schools and health care facilities, and a higher concentration of industries and hazardous wastes sites nearby (15). The siting of key sources of pollution located within or near Black neighborhoods results in another form of structural racism, i.e., environmental injustice, with residents in these communities experiencing a disproportionate burden of environmental exposures to contaminants in the air they breathe, water they drink, and where their children play (16–19).

Owing to critical gaps in our understanding of Black-White disparities in perinatal health, we established the Maternal and Infant Environmental Health Riskscape (MIEHR) Center, an NIH P50 Center of Excellence on Environmental Health Disparities Research, with an overall goal to evaluate the impact of multiple stressors on adverse maternal and infant outcomes in the greater Houston area. Premised on an environmental riskscape framework (20), we are examining chemical and non-chemical stressors in the biological, physical, social, and built environments that contribute to racial disparities in perinatal health, either directly or in combination with each other. Moreover, the study location provides a nexus for research on the impact of the environment on perinatal health disparities as Houston is the most diverse city in the nation (21) and is unfortunately also plagued by income disparities, with far greater of proportions of Hispanic (22%) and Black (20%) residents who live in poverty as compared with non-Hispanic white residents (5%) (22). In this paper, we describe the protocols being used in recruitment of the MIEHR cohort and provide exposure profiles for the cohort (and separately for Black and white participants) enrolled through February 28, 2023.

## Methods

### Recruitment

**Figure 1:**
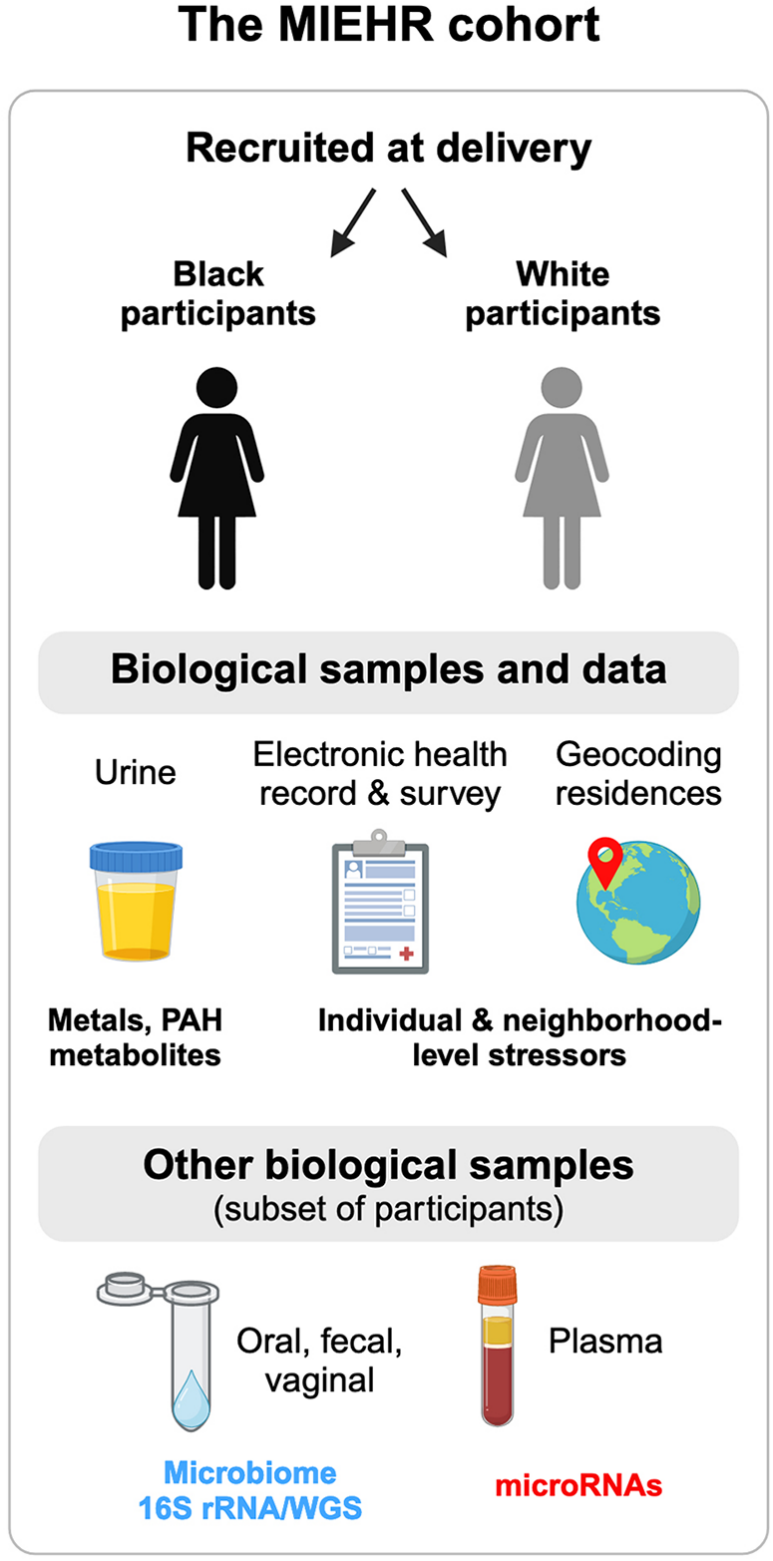
Overview of the MIEHR cohort recruited at major obstetric hospitals in Houston, Texas (April 2021-onwards) and the research projects underway. This image was created with BioRender.com.

The MIEHR cohort has a goal of recruiting ∼1,200 non-Hispanic Black and non-Hispanic white maternal-infant dyads from three large academic OB/GYN hospitals in the Texas Medical Center (TMC) in Houston, Texas (see Figure 1). Enrollment began in April 2021 at Memorial Hermann Hospital followed by enrollment at Ben Taub Hospital in July 2021 and at Texas Children's Pavilion for Women in June 2022. Eligibility criteria include the following: resident of the 8-county greater Houston area (Brazoria, Chambers, Fort Bend, Galveston, Harris, Liberty, Montgomery, or Waller County); 18 years of age or older; non-Hispanic Black/African American or non-Hispanic White, by self-identification; singleton delivery with no identified congenital anomaly; cognitively aware enough to participate in the study (i.e., able to provide informed consent); and English-speaker. The study protocol has been reviewed and approved by the IRBs at Baylor College of Medicine and The University of Texas Health Science Center at Houston under a reliance agreement.

Potential participants are initially identified using unified electronic health record (EHR) systems at each hospital enabling ready access, identification, and patient scheduling and tracking. Each weekday, trained obstetrics research coordinators review records of potential pregnant people who have been admitted for labor and delivery during the previous 24-hr period (or 72-hr period for Monday mornings) as well as antepartum and postpartum lists of patients. Potentially eligible participants are approached by research coordinators at a time when it does not interfere with their clinical care to verbally confirm eligibility. Potential participants who are interested and eligible (meeting inclusion and exclusion criteria) are assigned a study identifier and written informed consent is obtained.

### Questionnaire administration

Once consented, research coordinators administer a questionnaire electronically in REDCap, which is HIPAA–compliant and secure. The questionnaire seeks information about the following: maternal and paternal sociodemographics; residential history during pregnancy; pregnancy history and prenatal care; tobacco, alcohol, and other substance use during pregnancy; antibiotic and probiotic use during pregnancy; and maternal family health history. We also ask participants whether they are willing to be recontacted for participation in additional research activities for which they or their child may be eligible in the future and if yes, to provide their contact information. Data are also abstracted from EHRs including: maternal height and pre-pregnancy weight, insurance status, vaccination history during pregnancy, comorbidities and chronic diagnoses, prior pregnancy history, obstetric complications and diagnoses related to the index pregnancy, date of last menstrual period, dates of all ultrasounds received and associated fetal biometry (estimated fetal weight, head circumference, biparietal diameter, abdominal circumference), date of delivery, type of delivery (e.g., vaginal, cesarean), infant sex, infant anthropometry (head circumference, weight, length), and infant Apgar scores.

### Biological sample collection

All participants are provided the opportunity to provide urine and blood biospecimens. A subset of pregnant people at Ben Taub Hospital and Texas Children's Pavilion for Women are asked to also provide oral and vaginal swabs and fecal samples, as well as consent to collect oral and fecal/meconium from their infants. Blood samples are collected in 10 ml EDTA lavender top tubes, preferably during routine blood draws, and are immediately (within one hour) transported in coolers with frozen gel packs to the laboratory for processing. Spot urine samples are collected in sterile 100 ml urine specimen containers and stored with fecal samples and oral and vaginal swabs (if collected) in a cooler with a gel pack until they are transported to the laboratory on the same day that they are collected. Whole blood, plasma and urine samples are aliquoted in 1.5 ml sterile cryovials. All samples are stored at −80°C. Maternal oral swabs will undergo 16S ribosomal RNA (16S rRNA) and whole genome sequencing (WGS), microRNAs (miRNAs) will be profiled in plasma, and metals and monohydroxylated polycyclic aromatic hydrocarbons (OH-PAHs) metabolites will be measured in urine samples (see below). All other biological samples are being banked for use and analyses in future studies.

### Individual-level exposures to non-chemical stressors (discrimination, stress and social support)

As part of the questionnaire, we administered Krieger's Experiences of Discrimination (EOD) scale, a validated nine-item measure about lifetime experiences of unfair treatment in different settings that has demonstrated high internal consistency and test-retest reliability (23). Specifically, pregnant people are asked how many times they have ever experienced discrimination in the following situations: at school; getting a job; at work; getting housing; getting medical care; getting service in a store/restaurant; getting credit, bank loans or a mortgage; on the street or in a public setting; from the police or in the courts. Responses on the EOD are coded as 0 (“never”), 1 (“once”), 2.5 (“2–3 times”), and 5 (“4 or more times”) and summed to compute situation and frequency scores that range from 0 to 9 and 0 to 45, respectively, where higher scores indicate greater experiences of discrimination (23). We also ask questions assessing participant's perceptions of stress in their lives and during their pregnancy (not stressful, average stress, very stressful), as well as the level of support from the father of their babies and from families and friends (none, a little, a good amount, and an excellent amount). Lastly, because the greater Houston area is prone to weather-related and industrial disasters (24) and stressful life events have the potential to increase risks of adverse birth outcomes (25), we ask about experiences related to Hurricane Harvey (that resulted in catastrophic flooding in the Houston area in August of 2017 and thereafter), as well as the COVID-19 pandemic.

### Individual-level exposures to chemical stressors

Cotinine, a marker of tobacco smoke exposure and the following OH-PAH metabolites are being assessed in maternal urine samples: 1-hydroxynaphthalene (1-NAP), 2- hydroxynaphthalene (2-NAP), 2- hydroxyphenanthrene (2-PHE), 3-hydroxyphenanthrene (3-PHE), 4-hydroxyphenanthrene (4-PHE), combined 1/9- hydroxyphenanthrene (1/9-PHE), combined 2/3/9- hydroxyfluorene (2/3/9-FLUO), 1-hydroxypyrene (1-PYR), 3-hydroxybenzo[c]phenanthrene (3-BCP), 1-hydroxychrysene (1-CHRY), 6-hydroxychrysene (6-CHRY), and 1-hydroxybenz[a]anthracene (1-BAA). Extraction of PAH metabolites from urine was performed by liquid-liquid extraction followed by liquid chromatography-tandem mass spectrometry (LC-MS/MS) analysis (26). Briefly, urine samples were spiked with an isotopically labeled internal standard mixture and mixed with 1 ml of 0.5 M ammonium acetate buffer containing 200 units/ml of β-glucuronidase/sulfatase enzyme (MP Biomedicals, LLC, Solon, OH, USA). The urine samples were incubated overnight (∼16 h) at 37°C. Urine samples were then diluted by the addition of 2 ml of water followed by extraction using a mixture of 80% pentane: 20% toluene (v/v). PAH metabolites were chromatographically separated using a Waters Acquity I-Class UPLC system (Waters Corporation; Milford, MA, USA) connected with an Acquity UPLC BEH C18 column (50 × 2.1 mm, 1.7 µm, Waters; Milford, MA, USA). Identification and quantification of PAH metabolites was performed on an ABSCIEX 5,500 triple quadrupole mass spectrometer (Applied Biosystems; Foster City, CA, USA). Quality assurance protocols include analysis of two Standard Reference Materials (SRM 3,672, SRM 3,673) containing certified values for several PAH metabolites. HPLC grade water was used for sample/procedural blanks. We replaced urinary concentrations of PAHs below the limit of detection (LOD) with values of the LOD divided by √2 (27). To account for urine dilution, creatine concentrations were also measured, and urinary OH-PAH metabolite concentrations were adjusted for creatinine concentrations. Urinary concentrations of 40 metals are also being measured (Lithium, Beryllium, Vanadium, Chromium, Manganese, Cobalt, Nickel, Copper, Zinc, Arsenic, Selenium, Rubidium, Strontium, Molybdenum, Cadmium, Tin, Antimony, Tellurium, Cesium, Barium, Tungsten, Thallium, Lead, Uranium; in addition to 16 rare-earth metals) and will be reported on in the future.

### Neighborhood-level exposures to non-chemical and chemical stressors

Participants' residential addresses at delivery and during pregnancy are geocoded using ArcGIS Pro (version 3.1, Esri, Redlands, CA). We are developing several area-level measures and linking them with a mother's residential history to inform specific aspects of their social, built, and physical neighborhood environments. A few of these measures are discussed in more detail below.

#### Proximity to point sources of pollution

Given that disparities in residential proximity to industrial facilities based on race/ethnicity and socioeconomic position have been documented (28), we are constructing metrics that will allow us to evaluate exposure risks associated with living near point sources of air pollution. To date, we have accessed location information on all national and state Superfund sites in the 8-county study area (*n* = 46) (29) and computed residential distance (based on address at delivery) to the nearest site for MIEHR study participants. Future work will construct similar metrics related to proximity to major roadways and other point or area sources of pollution.

#### Tree canopy coverage

We computed the percentage of tree canopy surrounding a participant's residence using data from the National Land Cover Database (NLCD) tree canopy dataset for 2021 that provides the proportion of tree canopy within 30 × 30 m^2^ gridded cells. Using ArcGIS Pro's Zonal Statistics as Table Tool, we averaged the percentages of tree canopy of all cells in which the centroid of the cell was contained within a 300 m buffer of a mother's residence.

#### Socioeconomic deprivation

We used U.S. Census American Community Survey (ACS) five-year (2016–2020) estimates of socioeconomic and demographic variables to construct Area Deprivation Index (ADI) for all census tracts in the study area. ADI is a composite measure of neighborhood socioeconomic disadvantage that incorporates information on education, employment, income, poverty, household, and housing characteristics (30). We applied the R “Sociome” package to construct estimates that includes 15 original ACS variables for constructing ADI (the number of households without a telephone and the number of occupied housing units without complete plumbing were excluded from the computation) (31). Higher ADI scores indicate greater neighborhood deprivation.

#### Social vulnerability

We downloaded data for the social vulnerability index (SVI) from the Centers for Disease Control and Prevention (CDC)/Agency for Toxic Substances and Disease Registry (ATSDR). The SVI is a census tract-level composite metric comprised of 15 neighborhood characteristics in four domains (socioeconomic factors, household composition and disability, minority status and language, and housing type and transportation) and identifies communities at risk for public health emergencies related to natural and anthropogenic disasters (32). Higher SVI values indicate higher risk.

#### Racialized economic segregation

As proposed by Krieger et al. (33), we constructed the Index of Concentration at the Extremes (ICE) combined for race and income for all census tracts in our 8-county study area, using data from the U.S. Census ACS. ICE is a spatial measure of racialized economic segregation and here, we contrasted census-tract level differences between the proportions of high-income (>$100,000) non-Hispanic white persons and low-income (<$25,000) non-Hispanic Black persons. ICE has values ranging from −1 (areas of extreme economic and racial privilege) to 1 (areas of extreme economic and racial privilege).

#### Food access

We downloaded census-tract level indicators of food access for the 8-county study area from the USDA Food Access Research Atlas for 2019, including proportion of housing units that are without a vehicle and beyond ½ mile from a supermarket (34).

### Statistical analyses

We sought to characterize individual and neighborhood characteristics among pregnant people who enrolled in the MIEHR cohort. We calculated descriptive statistics for individual-level sociodemographic, behavioral, and health history information collected from questionnaires or abstracted from EHRs; data are presented both overall and by race. We also summarized the responses to the EOD scale and questions about sources of stress and social support by race. We computed summary statistics including the mean and standard deviation, selected percentiles, and detection frequency for urinary concentrations of selected OH-PAHs with at least 50% of values above the LOD (i.e., 1-NAP, 2-NAP, 2-PHEN, 3-PHEN, 2/3/9-FLUO and 1-PYR), as well as cotinine. Spearman rank correlation analysis was conducted between cotinine and OH-PAHs. Over the 8-county study area, we categorized values of ADI, ICE, and SVI into quintiles whereas we classified food access by tertiles because of a highly skewed distribution. We linked the census tract of a pregnant person's residence at delivery to the appropriate quantile of each metric and evaluated the percentile breakdown of neighborhood features for the study population together and stratified by race. Statistical or spatial analyses were performed in SAS (version 9.4) or ArcGIS (version 3.1.2).

## Results

As of February 28, 2023, 1,244 pregnant people were enrolled in the MIEHR cohort: 926 (74.4%) at Memorial Hermann Hospital, 211 (17.0%) at Texas Children's Pavilion for Women and 107 (8.6%) at Ben Taub Hospital. In total, nearly 80% of participants agreed to be re-contacted. Almost all participants (*n* = 1,241, 99.8%) provided urine samples and 595 (49.8%) provided blood samples. We also compared pregnant people who provided blood samples to the total cohort and there were little differences in the sociodemographic characteristics between these two groups. Among participants who were offered the opportunity to provide additional biological samples (*n* = 318), most (93.1%) provided oral swabs whereas relatively few provided vaginal swabs (24.2%) or fecal (15.1%) samples.

**Figure 2:**
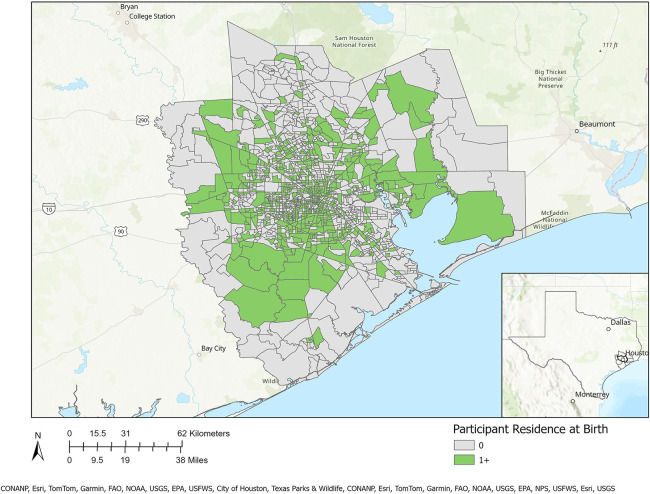
Residential locations of MIEHR cohort participants recruited through February 2023 in the greater Houston (8-county) study area (census tracts with residential locations of at least one study participant are shaded in green).

Table 1 presents a sociodemographic breakdown of the MIEHR cohort. Fifty-six percent of pregnant people were between the ages of 25–34 when they delivered their infants; most (61.1%) were non-Hispanic Black. Similar proportions of pregnant people report an annual household income of less than $35,000 (35.5%) or $75,000 or more (38.5%). Most pregnant people did not smoke (96%) or use alcohol (87.3%) during their pregnancy. There are notable differences in the sociodemographic profiles of Black and white pregnant people in the MIEHR cohort: 33.2% of Black pregnant people were less than 25 years of age when they delivered their infants as compared to 8.3% of white pregnant people; almost two-thirds (63.4%) of Black pregnant people were single as compared to 10.1% of white pregnant people; there was a five-fold difference in the percentage of Black pregnant people with household incomes lower than $35,000 as compared to white pregnant people; and a greater proportion of Black pregnant people as compared to white pregnant people initiated prenatal care at or after 13 weeks (21.1% vs. 6.8%). Regarding lifestyle factors, while the prevalence was low in both groups, there was almost a 3-fold increase in the proportion of white pregnant people as compared to Black pregnant people who reported using alcohol during their pregnancy (20.7% vs. 7.6%, respectively). As shown in Figure 2, the MIEHR cohort comes from a large, dispersed, geographic area in greater Houston. Over three-fourths of pregnant people (76.7%) did not move during their pregnancy. Among pregnant people who lived at more than one address while pregnant, 266 (21.4%) reported one move, 17 (1.4%) reported two moves, and 3 (0.2%) reported three moves.

**Table 1:** Sociodemographic characteristics of MIEHR study participants, greater Houston area, April 2021—February 2023, *N* = 1,244.

Sociodemographic Characteristics	Total	Black	White
	*N* = 1,244	*N* = 760	*N* = 484
	*n* (%)	*n* (%)	*n* (%)
Recruitment site			
Ben Taub Hospital	107 (8.6)	83 (10.9)	24 (5.0)
Texas Children's Pavilion for Women	211 (17.0)	82 (10.8)	129 (26.7)
Memorial Hermann Hospital	926 (74.4)	595 (78.3)	331 (68.4)
Age (years)			
<25	292 (23.5)	252 (33.2)	40 (8.3)
25–29	346 (27.8)	224 (29.5)	122 (25.2)
30–34	347 (27.9)	170 (22.4)	177 (36.6)
≥35	259 (20.8)	114 (15.0)	145 (30.0)
Preterm birth			
Pre-term	259 (20.8)	178 (23.4)	81 (16.7)
Full term	985 (79.2)	582 (76.6)	403 (83.3)
Nativity			
U.S.-born	1,137 (91.4)	692 (91.1)	445 (91.9)
Foreign-born	107 (8.6)	68 (8.9)	39 (8.1)
Employment			
Yes	749 (60.2)	386 (50.8)	363 (75.0)
Not employed	495 (39.8)	374 (49.2)	121 (25.0)
Highest educational attainment			
≤High school degree	408 (32.8)	351 (46.2)	57 (11.8)
College degree or higher	836 (67.2)	409 (53.8)	427 (88.2)
Marital status			
Single, never married	531 (42.7)	482 (63.4)	49 (10.1)
Married/Living with partner	696 (55.9)	265 (34.9)	431 (89.0)
Separated/widowed/divorced	17 (1.4)	13 (1.7)	4 (0.8)
Income			
Less than $ 34,999	441 (35.5)	398 (52.4)	43 (8.9)
$ 35,000–$ 74,999	254 (20.4)	194 (25.5)	60 (12.4)
$ 75,000 and above	479 (38.5)	105 (13.8)	374 (77.3)
Don't know	52 (4.2)	48 (6.3)	4 (0.8)
Prefer not to answer	18 (1.4)	15 (2.0)	3 (0.6)
Smoked cigarettes during pregnancy			
Never smoker	939 (75.5)	616 (81.1)	323 (66.7)
No	255 (20.5)	109 (14.3)	146 (30.2)
Yes	50 (4.0)	35 (4.6)	15 (3.1)
Exposure to secondhand cigarette smoke in the home or car during pregnancy			
No	1,015 (81.6)	581 (76.4)	434 (89.7)
Yes	228 (18.3)	179 (23.6)	49 (10.1)
Missing	1 (0.1)	0 (0.0)	1 (0.2)
Consumed alcohol during pregnancy			
Never drinker	202 (16.2)	181 (23.8)	21 (4.3)
No	883 (71.0)	521 (68.6)	362 (74.8)
Yes	158 (12.7)	58 (7.6)	100 (20.7)
Missing	1 (0.1)	0 (0.0)	1 (0.2)
Initiation of prenatal care			
No prenatal care	12 (1.0)	10 (1.3)	2 (0.4)
<13 weeks	998 (80.2)	553 (72.8)	445 (91.9)
≥13 weeks	193 (15.5)	160 (21.1)	33 (6.8)
Don't know	40 (3.2)	36 (4.7)	4 (0.8)
Prefer not to answer	1 (0.1)	1 (0.1)	0 (0.0)

### Individual-level exposures: non-chemical stressors (discrimination, stress and social support)

Table 2 reports on experiences of lifetime discrimination reported by pregnant people in different settings. In total, 83.9% of white participants reported no experiences of lifetime discrimination as compared to 47.4% of Black participants. The most common situations for Black participants reported experiencing discrimination were when they were getting services in a store or restaurant (32.5%), on the street or in a public setting (31.7%) or at work (30.5%). The summary scores for frequency of experiencing discrimination were 5.8 (SD = 8.7) and 0.9 (SD = 2.8) among Black and white participants, respectively. Table 3 summarizes stress experiences following the arrival of Hurricane Harvey in August 2017 and during the COVID-19 pandemic. A larger proportion of Black pregnant people (38.0%) than white pregnant people (16.9%) reported being impacted by Hurricane Harvey and had higher levels of stress in all contexts (i.e., new or worsened respiratory conditions; new or worsened anxiety; new or worsened depression; displaced from home; experienced extensive property loss or damage; or experienced new or worsened financial hardship). While more white than Black participants report that they or a family member tested positive for SARS-CoV-2, a greater proportion of Black participants reported that they (or a family member) were hospitalized. The financial impact of the COVID-19 pandemic in terms of employment (reductions in wages, hour worked or job loss) was greater among white participants (64.3%) as compared to Black participants (49.9%) whereas more Black than white participants had difficulty with getting food (15.3 vs. 7.9%), housing (13.3 vs. 2.7%) or transportation (11.6 vs. 2.9%).

**Table 2:** Experiences of discrimination of MIEHR study participants, greater Houston area, April 2021—February 2023.

Experiences of discrimination^a^	Black	White
	*N* = 760	*N* = 484
Number	*n* (%)	*n* (%)
0	360 (47.4)	406 (83.9)
1–2	163 (21.4)	57 (11.8)
3+	236 (31.1)	19 (3.9)
Missing	1 (0.1)	2 (0.4)
Summary score	mean ± SD	
Situations (possible range: 0–9)	1.8 ± 2.4	0.3 ± 0.6
Frequency (possible range: 0–45)	5.8 ± 8.7	0.9 ± 2.8

^a^ In 9 scenarios.

**Table 3:** Stress events during hurricane harvey (August 2017) and the COVID-19 pandemic, MIEHR study participants, greater Houston area, April 2021—February 2023.

Stress events	Black	White
	*N* = 760	*N* = 484
	*n* (%)	*n* (%)
Impacted by Hurricane Harvey		
No	470 (61.8)	400 (82.6)
Yes	289 (38.0)	82 (16.9)
Don't know	1 (0.1)	1 (0.2)
Missing	0 (0.0)	1 (0.2)
Tested positive for COVID-19		
No	501 (65.9)	232 (47.9)
Yes	257 (33.8)	251 (51.9)
Don't know	1 (0.1)	0 (0.0)
Missing	1 (0.1)	1 (0.2)
Hospitalized for COVID-19		
No	231 (89.9)	244 (97.2)
Yes	26 (10.1)	7 (2.8)
Someone in the household tested positive for COVID-19		
No	543 (71.4)	232 (47.9)
Yes	216 (28.4)	250 (51.7)
Don't know	0 (0.0)	1 (0.2)
Missing	1 (0.1)	1 (0.2)
Someone in the household hospitalized for COVID-19		
No	195 (90.3)	241 (96.4)
Yes	21 (9.7)	9 (3.6)
Reduction in reduced wages, work hours or lost job during the pandemic		
No	378 (49.7)	172 (35.5)
Yes	379 (49.9)	311 (64.3)
Don't know	2 (0.3)	0 (0.0)
Missing	1 (0.1)	1 (0.2)
Difficulty with childcare access during the pandemic		
No	628 (82.6)	397 (82.0)
Yes	128 (16.8)	86 (17.8)
Don't know	3 (0.4)	0 (0.0)
Missing	1 (0.1)	1 (0.2)
Difficulty getting food during the pandemic		
No	643 (84.6)	443 (91.5)
Yes	116 (15.3)	38 (7.9)
Don't know	0 (0.0)	2 (0.4)
Missing	1 (0.1)	1 (0.2)
Difficulty with housing during the pandemic		
No	657 (86.4)	469 (96.9)
Yes	101 (13.3)	13 (2.7)
Don't know	0 (0.0)	1 (0.2)
Prefer not to answer	1 (0.1)	0 (0.0)
Missing	1 (0.1)	1 (0.2)
Difficulty with transportation during the pandemic		
No	670 (88.2)	467 (96.5)
Yes	88 (11.6)	14 (2.9)
Don't know	0 (0.0)	2 (0.4)
Prefer not to answer	1 (0.1)	0 (0.0)
Missing	1 (0.1)	1 (0.2)
Difficulty getting medication, accessing healthcare, or paying for medical expenses during the pandemic		
No	670 (88.2)	456 (94.2)
Yes	89 (11.7)	25 (5.2)
Don't know	0 (0.0)	2 (0.4)
Missing	1 (0.1)	1 (0.2)

In contrast to experiences following Hurricane Harvey or during the COVID-19 pandemic, higher proportions of Black as compared to white pregnant people reported “not stressful” when asked about the amount of stress during their pregnancy (30.4% vs. 11.0%) whereas the proportions of participants reporting “very stressful” were similar between the groups (see Table 4). Also shown in Table 4 are summaries of responses about assistance and support from the father or family members and friends. Whereas 83.5% of white participants reported receiving an “excellent amount” of support from the father, only 57.5% of Black participants reported this same level of support. There were also differences by race for participants receiving low levels of social support with 17.5% of Black participants reporting “a little” or “none”, as compared to 5.4% of white participants. In contrast, there were modest differences by race in the amount of assistance and support from family members and friends.

**Table 4:** Stress and support during pregnancy, by race, of MIEHR study participants, greater Houston area, April 2021—February 2023.

	Black	White
	*N* = 760	*N* = 484
	*n* (%)	*n* (%)
Description of the amount of stress during pregnancy		
Not stressful	231 (30.4)	53 (11.0)
Average stress	335 (44.1)	294 (60.7)
Very stressful	193 (25.4)	136 (28.1)
Don't know	0 (0.0)	0 (0.0)
Prefer not to answer	0 (0.0)	0 (0.0)
Missing	1 (0.1)	1 (0.2)
The amount of assistance and support received during pregnancy from the baby's father		
None	77 (10.1)	16 (3.3)
A little	56 (7.4)	10 (2.1)
A good amount	182 (23.9)	49 (10.1)
An excellent amount	437 (57.5)	404 (83.5)
Don't know	1 (0.1)	1 (0.2)
Prefer not to answer	6 (0.8)	3 (0.6)
Missing	1 (0.1)	1 (0.2)
The amount of assistance and support received during pregnancy from family members or friends		
None	42 (5.5)	7 (1.4)
A little	51 (6.7)	25 (5.2)
A good amount	209 (27.5)	119 (24.6)
An excellent amount	456 (60.0)	331 (68.4)
Don't know	1 (0.1)	0 (0.0)
Prefer not to answer	0 (0.0)	1 (0.2)
Missing	1 (0.1)	1 (0.2)

### Individual-level exposures: chemical stressors

**Figure 3:**
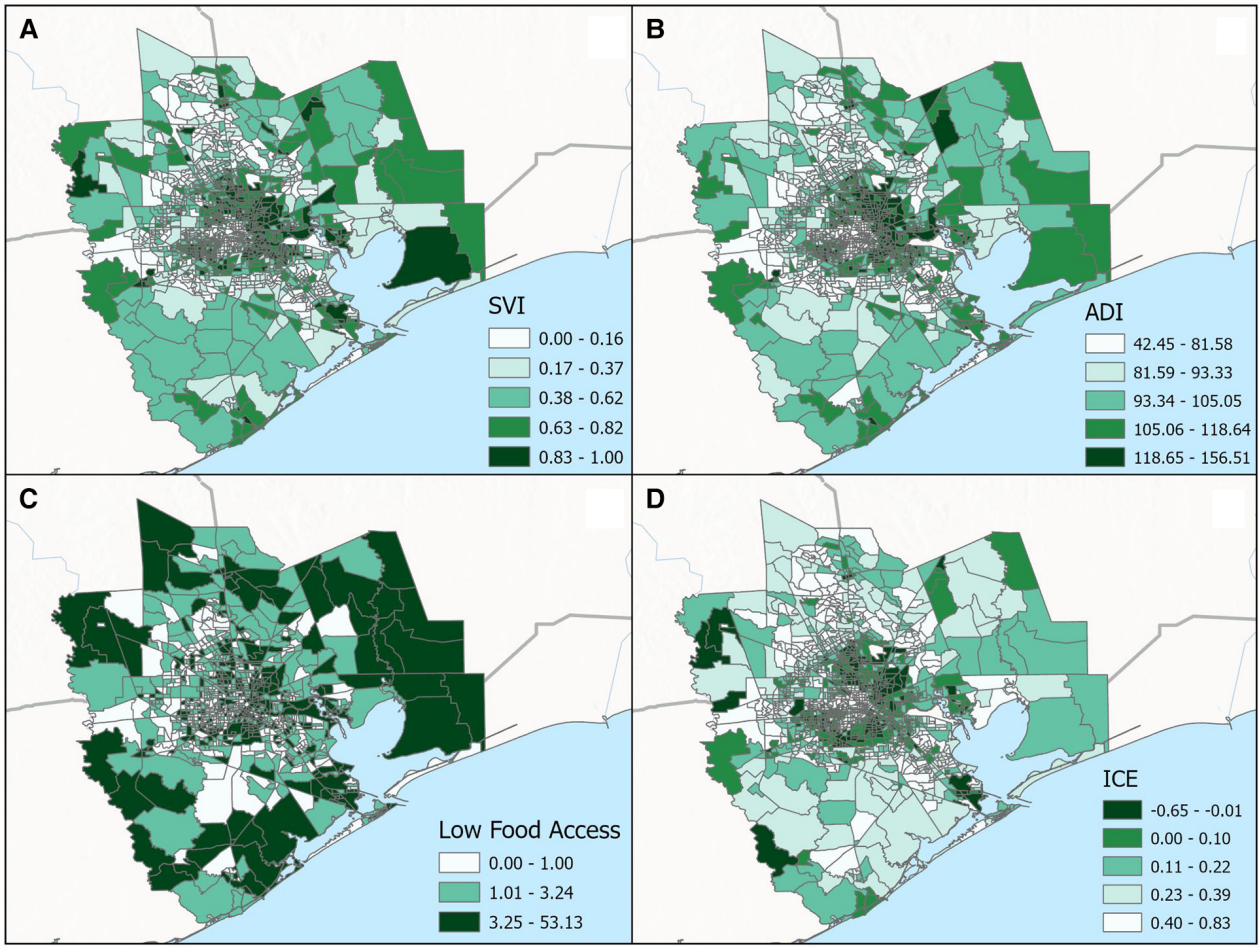
Categorical breakdown across census tracts for the (**A**) social vulnerability index (SVI), (**B**) area deprivation index (ADI), (**C**) low food access and (**D**) index of concentration at the extremes (ICE) for the greater Houston (8-county) study area.

Urinary concentrations of OH-PAH metabolites (μg/g creatinine) for 579 study participants showed that at least 50% of the values were above the LOD for 1-NAP (100%), 2-NAP (100%), 2/3/9-FLUO (83.8%), 1/9-PHEN (74.1%), 2-PHEN (58.2%), and 1-PYR (57.2%). The 50th (25th and 75th) percentiles for these PAH metabolites were 0.634 (0.384,1.107) (1-NAP), 5.844 (3.136, 10.595) (2-NAP), 0.032 (0.020, 0.053) (2-PHEN), 0.040 (0.023, 0.072) (1/9-PHEN), 0.070 (0.044, 0.127) (2/3/9-FLUO) and 0.036 (0.022, 0.062) (1-PYR) μg/g creatinine (Figure 3). Because PAHs are constituents of cigarette smoke, a heat map of the Spearman rank correlation coefficients for these OH-PAHs as well as cotinine was performed as shown in Supplementary Figure S1. Pair-wise correlations ranged from 0.085 to 0.690 and most (*n* = 14; 66.7%) of the correlation coefficients were 0.5 or lower. The highest correlations were observed between 2-PHEN and 1-PYY (0.69), 2-PHEN and 2/3/9-FLUOR (0.67), 1/9-PHEN and 2/3/9-FLUOR (0.66), 2-PHEN and 1/9-PHEN (0.65), 1/9-PHEN and 1-PYR (0.63), 2/3/9-FLUOR and 1-PYY (0.57) and 1-NAP and 2-NAP (0.54).

### Neighborhood-level exposures: Non-chemical stressors (tree canopy, socioeconomic deprivation, social vulnerability, residential segregation, food access)

**Figure 4:**
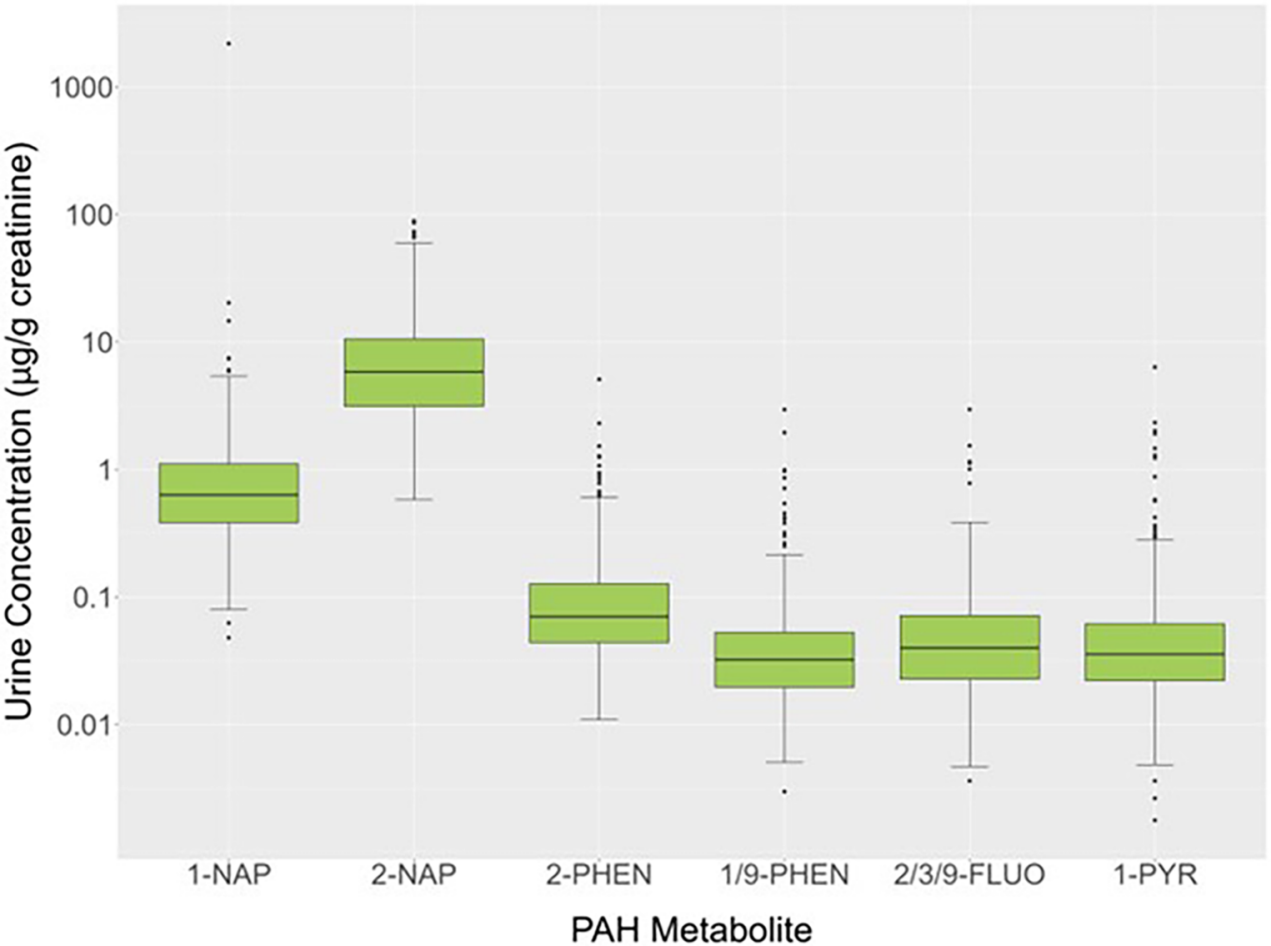
Box plots of OH-PAHS for a subset of the MIEHR cohort (*n* = 579).

In total, the median proportion of tree canopy cover within 300 m of participant's residence at delivery was 9%; 95% of participants were classified as having less than 23% tree canopy cover near their homes. There were little differences in this metric of residential greenness by race—the 25th, 50th, and 75th percentiles of tree canopy cover were 4.8, 9.4, and 15.4% for white participants and 4.8, 9.0 and 13.3% for Black participants. Figure 4 displays the spatial distribution of census tract-level ADI, SVI, ICE and Food Access for the 8-county study area. As shown in Table 5, substantially larger proportions of Black participants as compared to white participants lived in neighborhoods with: (1) high levels of socioeconomic disadvantage (upper two quintiles for ADI: 64.5% vs. 17.5%, respectively), (2) greater risk for public health emergencies (upper two quintiles for SVI: 59.8% vs. 19.6%, respectively), (3) higher levels of racialized economic segregation (lower two quintiles of ICE: 73.8% vs. 19.7%, respectively) and (4) the lowest levels of food access (upper tertile of food access: 49.7% vs. 16.7%, respectively).

**Table 5:** Neighborhood features of MIEHR study participants, greater Houston area, April 2021—February 2023.

Neighborhood feature	Total	Black	White
	*N* = 1,244	*N* = 760	*N* = 484
	*n* (%)	*n* (%)	*n* (%)
Area deprivation index (ADI)			
Q1 (42.5–75.64)	155 (12.5)	24 (3.2)	131 (27.1)
Q2 (75.65–89.98)	251 (20.2)	95 (12.5)	156 (32.2)
Q3 (89.99–104.96)	263 (21.1)	151 (19.9)	112 (23.1)
Q4 (104.97–121.56)	327 (26.3)	263 (34.6)	64 (13.2)
Q5 (121.57–156.51)	248 (19.9)	227 (29.9)	21 (4.3)
Social vulnerability index (SVI)			
Q1 (0–0.17)	240 (19.3)	53 (7.0)	187 (38.6)
Q2 (0.18–0.37)	224 (18.0)	102 (13.4)	122 (25.2)
Q3 (0.38–0.62)	231 (18.6)	151 (19.9)	80 (16.5)
Q4 (0.63–0.82)	257 (20.7)	199 (26.2)	58 (12.0)
Q5 (0.83–1.00)	292 (23.5)	255 (33.6)	37 (7.6)
Index of concentration at the extremes (ICE _(race__ + income)_)			
Q1 (−0.65 - −0.01)	456 (36.7)	418 (55.0)	38 (7.9)
Q2 (0–0.10)	200 (16.1)	143 (18.8)	57 (11.8)
Q3 (0.11–0.22)	182 (14.6)	108 (14.2)	74 (15.3)
Q4 (0.23–0.39)	187 (15.0)	55 (7.2)	132 (27.3)
Q5 (0.40–0.83)	219 (17.6)	36 (4.7)	183 (37.8)
% Low food access to supermarkets			
Q1 (0–1.00)	416 (33.4)	167 (22.0)	249 (51.5)
Q2 (1.01–3.24)	369 (29.7)	215 (28.3)	154 (31.8)
Q3 (3.25–53.13)	459 (36.9)	378 (49.7)	81 (16.7)

### Neighborhood-level exposures: chemical stressors (proximity to superfund sites)

The median value from a participant's residence to the closest Superfund site was 3.62 miles, with the residences of Black participants slightly closer to a site (3.40 miles) as compared to residences of white participants (3.90 miles). The interquartile range of residential distances to the nearest Superfund site was 3.61 miles for all participants, and 3.38 and 4.05 miles for Black and white participants, respectively.

## Discussion

Black pregnant people suffer the highest risks of poor pregnancy outcomes in the nation and the reasons for this disparity are poorly understood. Hence, we established the MIEHR cohort in a large and diverse urban area in the U.S. to unravel factors that help to explain Black-White disparities in preterm birth and other perinatal outcomes. Our focus is on examining effects of chemical and non-chemical stressors in the biological, physical, social, and built environments, i.e., the environmental riskscape, which contribute to racial disparities in maternal and child health. Extensive data is being collected in the MIEHR cohort through administration of questionnaires and EHR abstraction, along with collection of biological samples for chemical, miRNA, and microbiome assessments. Beyond individual-level factors, features of a pregnant person's neighborhood environment are also being characterized. Initial analysis of individual- and neighborhood-level factors among the 1,244 pregnant people enrolled in MIEHR through the end of February 2023 suggests differences between Black and white pregnant people in experiences of discrimination, stress, and levels of support, as well as in characteristics of their neighborhoods.

An earlier meta-analysis of the epidemiologic evidence reported significant albeit relatively small impacts of individual-level sources of psychosocial stress on adverse birth outcomes (35). Stress during pregnancy is associated with increased concentrations of catecholamines (36) and activation of the hypothalamic-pituitary-adrenal (HPA) axis that triggers a cascade of events culminating in the release of cortisol (35, 37), which crosses the placenta and may adversely impact fetal development and parturition (37). Our findings regarding racial differences in stress levels depended on whether questions were specific to events (like Hurricane Harvey or the pandemic) or were general in nature. In the aftermath of specific disasters, Black participants reported experiencing higher levels of stress than white participants, while reports of general stress were lower among Black as compared with white participants. Findings from the literature have been mixed. In one study and contrary to our findings, perceived stress levels, as assessed using Cohen's Perceived Stress Scale (PSS-14 ≥ 30), were greater among Black (24.7%) than white (7.7%) pregnant participants from Philadelphia, PA (38). On the other hand in a study using the Pregnancy Risk Assessment Monitoring System (PRAMS) data from 2012 to 2013, the prevalence of traumatic stressors were higher among white participants as compared to Black participants whereas there were little differences for either financial or relationship stressors (39).

A recent review points to a greater role for stressors like racial discrimination on increased risks for adverse birth outcomes (40). Consistent with prior findings that individuals of color have greater opportunity to experience stressful conditions due to the intersection of race and gender (41), Black pregnant people in the MIEHR cohort experienced greater discrimination as compared to their white counterparts. Our findings are similar to results from an earlier investigation of 112 pregnant people who were recruited in Chicago, Illinois that used the same scale as we applied in our study (42), as well as in a recently published cross-sectional analysis of 198 women that relied on a different tool (the Schedule of Racist Events measure) to assess discrimination (43).

Whereas there were modest differences in levels of support from family members and friends for Black and white pregnant people in our study, white pregnant people generally reported receiving higher levels of paternal support. The benefits of social support are hypothesized to operate through several pathways by reducing inflammation and biological aging. Population-based studies have reported Black-White differences in biological aging (44, 45), as well as inverse associations among Black (but not white) adults who participate in more social groups (44). A pilot study of 49 pregnant Black participants reported inverse associations between social support and pro-inflammatory cytokines (IL-2, IL-5, andIL-6) (46). While results from a systematic review and metanalysis suggest associations between low social support and increased risks for preterm birth, especially among participants with high stress levels (pooled OR of 1.52 (95% CI, 1.18, 1.97) (47), a consensus document from the March of Dimes concluded the evidence was insufficient regarding the role of social support in explaining Black-white disparities in preterm birth (48).

Neighborhoods represent shared physical characteristics, social and economic resources, and social interaction among residents (49, 50). Neighborhood socioeconomic disadvantage, which is a well-studied attribute of the neighborhood environment, has been consistently associated with adverse perinatal health even after controlling for individual-level factors (49, 51–53). Moreover, consistent with the hypothesis of a psychosocial pathway through which the residential environment adversely impacts pregnant people (54), studies have found in non-pregnant populations that neighborhood conditions associated with disadvantage are conducive to stress (55) and are linked to increased cumulative biological risk, allostatic load and cortisol levels (56–59). In our study, we found substantially larger proportions of Black participants as compared to white participants lived in neighborhoods with high levels of socioeconomic disadvantage. Similarly, based on assessment of ICE and SVI, higher proportions of Black women lived in neighborhoods that were socially and racially isolated or at elevated risk for natural or industrial disasters, respectively. While the evidence for the impact of residential greenspace on perinatal health is mixed (60), we are also computing metrics of greenness surrounding homes that a participants lived in during their pregnancy, including at delivery. Not surprisingly in an urban area such as Greater Houston, on average, there was less than 10% of tree canopy near a participant's residence and we found little differences in residential greenness between Black and white participants.

Given inequalities in the spatial distribution of environmental hazards, disadvantaged communities experience a higher burden of exposure to chemical stressors as evidenced in studies conducted across the U.S (17, 61, 62)., as well as in large urban areas (63, 64) including Houston (65). Hence, our focus on factors in the environmental riskscape extends to such stressors, particularly exposures to metals and PAHs in the physical environment that can occur via multiple pathways (ingestion, inhalation, or skin contact). Oxidative stress is a common pathway for metal-induced physiologic perturbations and subsequent toxicities (66, 67) and has been implicated in PAH toxicity as well (68, 69). During pregnancy, oxidative stress may result in alterations in signaling pathways, protein modifications, activation of inflammatory pathways and DNA oxidation; all of which may impact vascular function at the maternal placental interface (70).

Comparison of measured urinary concentrations of OH-PAHs in the present investigation with those previously reported in other populations, either during pregnancy or around the time of delivery is limited given differences in adjustment for urine dilution; as such, our comparisons were restricted to studies where OH-PAH concentrations were adjusted for creatinine. Median 1-PYR concentrations in our study (0.036 μg/g creatinine) were similar to levels measured in investigations conducted on pregnant people in Brazil (0.030 μg/g creatinine) and Saudia Arabia (0.050 μg/g creatinine) whereas they were lower than previously reported in studies from the Czech Republic (0.120 μg/g creatinine) (71), Japan (0.124 μg/g creatinine) (72), Poland (0.35 μg/g creatinine) (73), Haojiang, China (0.570 μg/g creatinine) (74), Taiyuan, China (1.83 μg/g creatinine) (75) and Iran (6.5 μg/g creatinine) (76). For 1-NAP, levels measured in the MIEHR cohort (median = 0.630 μg/g creatinine) fell between those reported in other studies. Whereas lower values were reported for pregnant people living in the Czech Republic (0.40 μg/g creatinine) (71), 1-NAP values were considerably higher in investigations in Iran (4.6 μg/g creatinine) and Brazil (16.99 μg/g creatinine) (77). For 2-NAP, concentrations were higher in our study when compared to levels in pregnant people in South Korea (78) [arithmetic mean (AM) = 9.44 μg/g creatinine vs. 0.010 μg/g creatinine], Canada [geometric mean (GM) = 6.002 μg/g creatinine vs. 2.61 μg/g creatinine] (79), Brazil (median = 5.84 μg/g creatinine vs. 3.62 μg/g creatinine vs.) or Iran (median = 5.84 μg/g creatinine vs. 2.5 μg/g creatinine). In contrast, median levels of 2-PHEN of 0.032 μg/g creatinine in the MIEHR cohort were low relative to reports in the Czech Republic (71) (0.170 μg/g creatinine), China (80) (0.109 μg/g creatinine), or Poland (73) (0.430 μg/g creatinine). Overall, PAH exposure patterns varied in our cohort compared to pregnant people in other countries; also, where comparisons could be made, concentrations in our study were similar (2-NAP) or lower (1-NAP and 1-PYR) than those reported for NHANES for either females ages 3 and older or adults ages 20 and older (81).

### Future directions

We continue to enroll pregnant people in the MIEHR cohort. Analyses of urinary metal concentrations are underway as are maternal oral microbiome testing and miRNA analyses in plasma samples. Work is also ongoing to characterize a participant's neighborhood environment more fully by developing metrics for proximity to major roadways and other major pollution sources. With complete cohort data, we will formally evaluate differences in exposure profiles between Black and white cohort members and associations between exposure to the mixture of metal and OH-PAH metabolites and perinatal health outcomes, as well as the potential modifying role of neighborhood stressors on these associations. We are also planning on developing disparity-aware classifiers to identify the most informative set of features that predict risk for preterm birth for Black and white women. Future studies will continue to follow up pregnant participants and their children to evaluate the impact of the environmental riskscape on their longer-term health and well-being.

## Data Availability

The datasets presented in this article are not readily available because de-identified data will be made available according to the restrictions as specified in the IRB protocol. Requests to access data should be directed to elaine.symanski@bcm.edu.
