# Supplementary material for: The Maternal and Infant Environmental Health Riskscape study of perinatal disparities in greater Houston: rationale, study design and participant profiles
Source: Front Reprod Health. 2024 Apr 22;6:1304717. doi: 10.3389/frph.2024.1304717 (PMC11070492; doi:10.3389/frph.2024.1304717)
Supplement: Supplementary file 1 [file Datasheet1.pdf]

The Maternal and Infant Environmental Health Riskscape study of perinatal disparities in greater Houston: rationale, study design and participant profiles

Elaine Symanski[1,2*], Kristina W. Whitworth[1,2], Hector Mendez-Figueroa[3], Kjersti M. Aagaard[4], Iman Moussa[1], Juan Alvarez[1], Adrien Chardon Fabian[1], Kurunthachalam Kannan[5], Cheryl L. Walker[1,6], Cristian Coarfa[1,6], Melissa A. Suter[4], and Hamisu M. Salihu[7]

1. Center for Precision Environmental Health, Baylor College of Medicine, Houston, TX, United States
2. Section of Epidemiology and Population Sciences, Department of Medicine, Baylor College of Medicine, Houston, TX, United States
3. Division of Maternal-Fetal Medicine, Department of Obstetrics, Gynecology and Reproductive Sciences, McGovern Medical School at UTHealth, Houston, TX, United States
4. Division of Maternal-Fetal Medicine, Department of Obstetrics & Gynecology, Baylor College of Medicine & Texas Children's Hospital, Houston, TX, United States
5. New York State Department of Health, Wadsworth Center, Albany, NY, United States
6. Department of Molecular and Cell Biology, Baylor College of Medicine, Houston, TX, United States
7. Department of Family and Community Medicine, Baylor College of Medicine, Houston, TX, United States

Corresponding author

Elaine Symanski, PhD
Professor, Center for Precision Environmental Health
Department of Medicine
Baylor College of Medicine
6550 Fannin St, Neurosensory NB315
Houston, Texas 77030-3411
elaine.symanski@bcm.edu

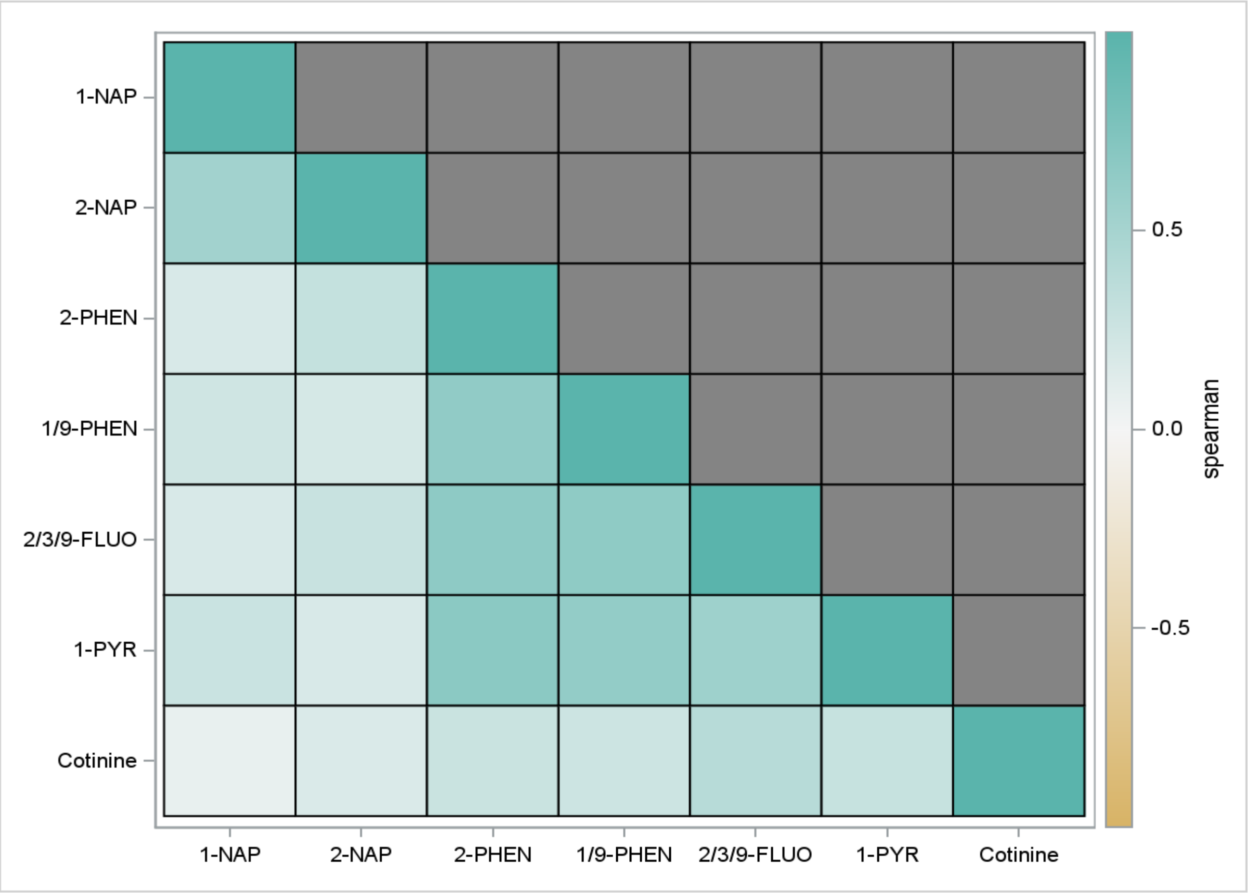

Supplemental Figure 1. Heat map of correlation coefficients between OH-PAH metabolites and cotinine.
